# The Relationship Between Radiotherapy-Induced Pain Response Score and Pain Biomarkers TRPV1, β-Endorphin (bEP), Neurotensin (NT), and Orexin A (OXA) in Patients with Bone Metastases

**DOI:** 10.3390/life15091372

**Published:** 2025-08-28

**Authors:** Sema Yilmaz Rakici, Adnan Yilmaz, Sibel Mataraci Karakas

**Affiliations:** 1 Department of Radiation Oncology, Faculty of Medicine, Recep Tayyip Erdogan University, 53100 Rize, Türkiye; 2 Department of Biochemistry, Faculty of Medicine, Recep Tayyip Erdogan University, 53100 Rize, Türkiye; adnan.yilmaz@erdogan.edu.tr (A.Y.); sibel.karakas@erdogan.edu.tr (S.M.K.)

**Keywords:** bone metastasis, radiotherapy, pain palliation, pain rating scales, biochemical biomarkers, TRPV1, β-endorphin, Neurotensin, Orexin A, neutrophil–leukocyte ratio (NLR)

## Abstract

**Objective:** Pain response scores were evaluated by associating pain biomarkers with several parameters affecting radiotherapy (RT)-induced pain response in patients with bone metastases. **Methods:** A newly developed ‘revised pain and response scale’ based on standardized scales was used for pain scoring. TRPV1, β-endorphin (bEP), neurotensin (NT), and orexin A (OXA) biomarkers were determined by ELISA before and after RT. **Results:** Pain response rates were 44.75% (*n* = 47) poor response, 10.5% (*n* = 11) moderate response, 44.75% (*n* = 47) good response. NLR before RT was higher in patients with poor response than those with good response (4.0 (1.3–36.7) vs. 2.6 (1.2–11.4), respectively (*p* = 0.036). NLR after RT was lower in patients with good response than in patients with poor response (3.1 (1.2–10.8) and 3.9 (0.8–37.2), respectively (*p* = 0.047). There was a significant correlation between response scores and NT, bEP, and TRPV1. In patients with good response, NT and bEP decreased, while TRPV1 increased, both of which were significant. Pre-RT and post-RT values were, respectively, NT: 631.4 (39.7–2863.0) vs. 400.3 (79.1–1479.0) *p* = 0.006) and bEP: 92.1 (18.7–228.8) vs. 49.1 (13.3–135.6) *p* ≤ 0.001). TRPV1 values: 321.7 (48.1–1100.7) vs. 352.8 (119.3–1510.9) *p* ≤ 0.001). **Conclusions:** The study found no difference in pain response scores between the different fractionation treatments. Significant changes in NT, bEP, and TRPV1 levels were seen in patients with a ‘good response’. Pain response ratings were potentially least affected by OXA. Changes in NT, TRPV1, and bEP levels represent RT’s pain response efficacy and patients’ pain perception. These pain biomarkers may be included in guidelines as part of pain response monitoring strategies in the future.

## 1. Introduction

Cancer-related bone metastases are a significant global health problem that develop in many advanced cancer cases [1]. When cancer cells migrate from their original sites to the bones, it marks the beginning of a serious problem known as bone metastasis. Bone metastases can cause pain that can severely affect a person’s quality of life [2]. The prevalence of pain in cancer patients is between 33% and 64%, depending on the stage of the disease [3]. Accurate scoring of pain is the first step towards effective and personalized treatment. The most commonly used standardized scales are ‘visual analog scale’ (VAS), ‘Verbal Rating Scale (VRS), Numeric Rating Scale’ (NRS), and ‘Faces Pain Scale’ (FPS) [4,5,6]. There are some biochemical biomarkers concerning cancer-related pain and its mechanisms. These are Transient Receptor Potential Cation Channel Subfamily V, Member 1 (TRPV1), βeta-endorphin (bEP), Neurotensin (NT), and Orexin A (OXA) [7,8,9].

RT, a common treatment modality for managing bone metastases, affects both biochemical markers and blood parameters [10,11]. While aiming to shrink the tumor and alleviate pain, the initial tissue response to radiation may cause inflammation and transient increases in markers [11]. RT reduces tumor burden and pain levels. This reduction involves complex interactions between biochemical pain markers and blood parameters. Biochemical markers significantly influence pain management strategies in cancer patients with bone metastases, and we believe that a comprehensive understanding of these variables is required to optimize treatment [12]. Pain-related biomarkers may interact in a variety of ways to alter pain perception and modulation, thereby determining the potential for therapeutic targets in pain management strategies.

Understanding the interactions between pain and its treatment in patients with bone metastases through biochemical markers can provide information on pain modulation and predict treatment responses, thus promoting personalized therapy strategies. Therefore, these biomarkers can guide optimization of a combination of treatment strategies, reflecting the actual changes in pain perception of clinical trial results.

## 2. Methods

The study was conducted prospectively in the Radiation Oncology Clinic of Recep Tayyip Erdogan University with the approval of local ethics committee (Approval No: E-40465587-050.01.04-1190 and date: 22 August 2024). The financial resources required for the study were provided by Recep Tayyip Erdogan University Scientific Research Projects Coordination Office (TSA-2023-1466).

### 2.1. Patient Selection and Radiotherapy

**Figure 1:**
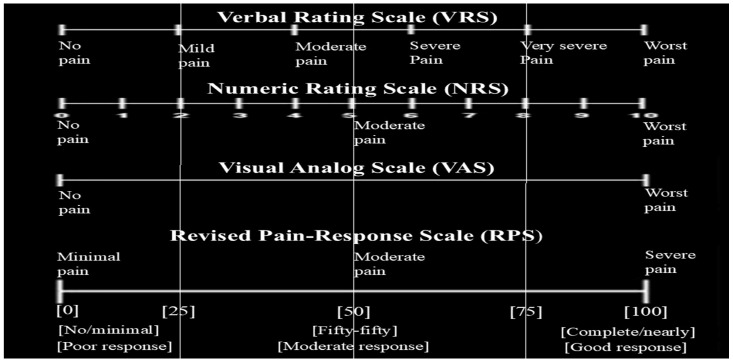
Pain and treatment response rating scales. From top to bottom: Verbal Rating Scale (VRS), Numeric Rating Scale (NRS), Visual Analog Scale (VAS), and the newly developed Revised Pain-Response Scale.

The study included 105 patients. A new ‘revised pain and response scale’ based on standardized scales such as VRS, NRS, VAS, and FPS was developed and used for the study. Figure 1 shows the comparison of the scales used in clinics and the newly created scale. Pre-treatment pain intensity and post-treatment pain response were measured on the same scale using standard scales. The pain score defined in scales such as VRS, NRS, VAS, and FPS for scores of 0, 1, and 2, or 0%, 15%, and 25%, classified as “mild-minimal pain” (Figure 1, first column). Moderate pain, severe pain, or scores of 3, 4, 5, 6, and 7, or scores between >25% and <75% (average 50%), are classified as “moderate pain” (Figure 1, middle two columns). Very severe pain and worst pain, or scores of 8, 9, and 10, or >75%, were classified as “severe pain” (Figure 1, last column). The degree of pain response after treatment was considered a “poor response” if there was minimal or no response or a reduction of up to 25%; a “moderate response” if the pain reduction was fifty-fifty or >25% to <75% (average 50%); and a “good response” if the pain was reduced completely, nearly completely, or by >75% (Figure 1).

The study included 105 patients. A new ‘revised pain and response scale’ based on standardized scales [13] such as VRS, NRS, VAS, and FPS were developed and used for the study. Figure 1 shows the comparison of the scales used in clinics and the newly created scale. Pre-treatment pain intensity and post-treatment pain response were used on the same scale by utilizing standard scales. The pain score defined in scales such as VRS, NRS, and VAS for scores 0, 1, and 2 or 0%, 15, and 25% or no pain and mild pain was classified as “mild-minimal pain”(Figure 1, first column). Moderate pain, severe pain, or scores of 3, 4, 5, 6, and 7, or scores in the >25% to <75% (average 50%), are classified as “moderate pain” (Figure 1, middle two columns). Very severe pain and worst pain or scores of 8, 9, and 10 or >75% were classified as “severe pain” (Figure 1, last column). The degree of pain response after treatment was considered a “poor response” if there was minimal or no response or a reduction of up to 25%, a “moderate response” if the pain reduction was fifty-fifty or >25% to <75% (average 50%), and a “good response” if the pain was reduced by completely, nearly completely, or by >75% (Figure 1).

Bone metastasis information was obtained from positron emission tomography (PET) and computed tomography (CT), SUV-max values from Gallium-68 prostate-specific membrane antigen (Ga-PSMA)-PET for prostate cancer, and fludeoxyglucose F18 (FDG)-PET reports for other cancer types were used. According to the number of bone metastases, oligometastasis was accepted if 4 or fewer, and multiple metastases were accepted if more than 4. Based on location, metastases were classified as axial (there was no calvarial bone in the study) if they were vertebral and vertebra–costal; appendicular if they originated from the pelvis and extremities; and mixed if they were present on both sides. In addition, according to the composition of the metastasis, it was classified into five groups: soft tissue lytic/destructive, PET CT involvement only, sclerotic, lytic, and mixed (if it contains more than one component).

PET-CT fusion was used to ensure the necessary precision in drawing the RT target tumor volumes of the patients. Different daily fraction sizes and total doses used according to the institutional protocol are shown in Table 1. In the treatment, conformal or IMRT plans were made using Elekta and Varian brand linear accelerator devices and treatment planning systems and radiotherapy treatments were applied (Elekta: Synergy model, Monto Carlo Medical System, Monaco version 5.51.10 Elekta AB, Stockholm, Sweden, Varian: Trilogy IX, Varian Medical Systems, Eclipse Version: 13.6 Varian, Palo Alto, CA, USA). Elekta: 600 MU/min, Varian: 400 MU/min maximum variable dose rate was selected. 6 MV photon beams were used in both devices [14,15].

### 2.2. Determination of Biochemical Pain Biomarkers

TRPV1 (Cat. No: SEF839Hu), bEP (Cat. No: CEA806Hu), NT (Cat. No: CEB203Hu), and OXA (Cat. No: CEA607Hu) levels in serum samples obtained before and during the first week following RT were determined using commercially obtained enzyme-linked immunosorbent assay (ELISA) kits from USCN. Microplates were coated with antibodies specific for the target biomarker. Samples were reacted with biotin-conjugated antibody and HRP-conjugated avidin. The color produced by the addition of TMB (3,3′,5,5′-Tetramethylbenzidine) substrate was measured spectrophotometrically at a wavelength of 450 nm. Biomarker concentrations were calculated in pg/mL by comparing the optical density (OD) values of the samples with the standard curve.

### 2.3. Statistical Analysis

Data were analyzed using the SPSS (Version 22 for Windows, SPSS Inc., Chicago, IL, USA) software package. Data were expressed as mean ± standard deviation and/or median (minimum and maximum value) for continuous variables. Frequency data were expressed as numbers and percentages (%). In statistical analyses, the conformity of continuous variables to normal distribution was evaluated by the ‘Kolmogorov–Smirnov Test’ and the ‘Shapiro–Wilk Test’ when necessary. The ‘Pearson Chi-square Test’ was used to compare frequency data. Since continuous variables did not conform to normal distribution, the ‘Kruskal–Wallis Test’ was used for intergroup comparisons. The ‘Wilcoxon Test’ was used to compare the data before and after RT in each group. Survival analyses were performed with the Kaplan–Meier test. In all statistical comparisons, *p* < 0.05 was considered statistically significant.

## 3. Results

Patient and tumor characteristics and RT response data are summarized in Table 1. A total of 105 patients were included in the study. The mean age of the patients was 65.5 ± 12.3 (35–87) years, and patients with poor response were younger than patients with good response (63.1 ± 13.1 vs. 67.7 ± 12.2). The male-to-female ratio was 1.5, and there was no difference in pain response rate between genders. Poor, moderate, and good response rates were, respectively, 44.75% (*n* = 47, seven patients had no response), 10.5% (*n* = 11), and 44.75% (*n* = 47, 18 patients had a complete response).

Patients with severe pain scores before treatment had ‘poorer response’ (*p* = 0.001). There was no significant correlation between the duration from the date of diagnosis and the development of metastasis, which ranged from 3 months to 60 months, and RT-induced pain score (*p* = 0.670). However, as the duration extended from 3 months to 60 months, the “good response” rate decreased from 61.7% (*n* = 29) to 8.5% (*n* = 4) (Table 1).

A good pain response rate was higher in patients with breast and prostate cancer (*p* = 0.043). There was no difference in pain response score in terms of the presence of perineural invasion (PNI), and whether the metastasis was vertebral, extremity, or mixed. When the metastases were compared in terms of lytic, destructive, soft tissue component, and PET uptake only, good pain palliation was achieved more in sclerotic metastases (*p* = 0.001).

There was no correlation between SUVmax values of the primary tumor, involved lymph node, or bone metastasis and pain response scores. However, SUVmax values of patients with poor pain response were higher than those with good response. Patients with SUVmax values above 10 had a poorer response score.

No statistically significant correlation was found between RT-related variables (previous RT, RT interval (“time between the start and end of RT (days)”), RT total dose, fraction dose, size of the target volume, percentage of dose covering the target volume, RT dose rate, Monitor unit (MU) and RT technique and post-treatment pain response scores.

The relationship between complete blood count (CBC), routine biochemical tests, and pain biomarkers with pain response score is shown in Table 2. There was no significant difference between Neu values and pain score groups before RT. After RT, Neu decreased statistically significantly in patients with good and poor responses. Compared to pre-RT, post-RT Neu values decreased in all patients regardless of good, moderate, or poor pain response: 4.6 (1.5–19.1) vs. 3.9 (0.8–25.0), *p* = 0.006 in poor response vs. 3.7 (1.4–11.7) vs. 3.0 (0.7–8.0) in good response (*p* ≤ 0.001). Neu decreased more in patients with good response compared to patients with poor response after RT (3.9 (0.8–25.0) vs. 3.0 (0.7–8.0), *p* = 0.010).

Leu was statistically significantly decreased in all patients after RT compared to pre-RT; poor and good response rates were 1.2 (0.2–2.0) vs. 0.9 (0.2–4.7) (*p* < 0.001) and 1.3 (0.3–3.3) vs. 0.8 (0.3–3.5) (*p* < 0.001).

Neu/Leu ratio (NLR) was found to be statistically significantly lower post-RT than pre-RT. Before RT, NLR was 4.0 (1.3–36.7) in patients with poor response and 2.6 (1.2–11.4) in patients with good response (*p* = 0.036). NLR after RT was lower in patients with good response than in patients with poor response (3.1 (1.2–10.8) vs. 3.9 (0.8–37.2) *p* = 0.047, respectively).

PLT was statistically significantly lower after RT than before RT for all pain scores. ALP and LDH values were not significantly different before and after treatment. ALP was lower after RT than before RT. There was no correlation between elevated tumor markers before RT and pain palliation.

The relationship between changes in biomarkers before and after RT and post-treatment pain scores is summarized in Table 3. When the relationship between NT, bEP, TRPV1, OXA, and response score was examined, a significant correlation was found in all except OXA. NT and bEP decreased after RT compared to pre-RT in patients with good response: NT 631.4 (39.7–2863.0) vs. 400.3 (79.1–1479.0), *p* = 0.006; and bEP 92.1 (18.7–228.8) vs. 49.1 (13.3–135.6), *p* ≤ 0.001. TRPV1 increased: 321.7 (48.1–1100.7) vs. 352.8 (119.3–1510.9), *p* ≤ 0.001. OXA increased, but the difference was not statistically significant: 1449.0 (492.0–3311) vs. 1589.5 (694.0–3703.0), *p* = 0.125. In patients with good response, the decrease in NT and bEP, and the increase in TRPV1 were statistically significant (Table 3).

The relationship between routine CBC and biochemical parameters before and after RT and pain biomarkers is summarized in Table 4. There is a positive correlation between Neu and TRPV1 change before RT (r = 0.20, *p* = 0.04). A significant negative correlation was found between the patients’ pre-RT Leu and PLT values and the change in post-RT bEP and OXA values: Leu with bEP (r = −0.23, *p* = 0.02), Leu with OXA (r = 0.20, *p* = 0.04), PLT with bEP (r = −0.22, *p* = 0.03), and PLT with OXA (r = 0.27, *p* = 0.01). No significant correlation was found between pre-RT LDH, ALP, and NLR and post-RT pain markers.

When the relationship between the change in routine blood tests before- RT and post-RT biochemical pain markers was analyzed, a statistically significant negative correlation was found between NLR and NT (r= −0.27, *p* = 0.03) (Table 4).

**Table 4:** Changes in hematological and biochemical routine blood tests and pain biomarkers before and after radiotherapy.

Pre-RT Parameters	Neurotensin		β-Endorphin		TRPV1		Orexin A	
	r	*p*	r	*p*	r	*p*	r	*p*
NEU	−0.08	0.40	−0.18	0.06	0.20	0.04	0.13	0.20
LEU	−0.02	0.86	−0.23	0.02	−0.05	0.59	0.20	0.04
PLT	0.10	0.29	−0.22	0.03	−0.05	0.59	0.27	0.01
LDH	−0.11	0.28	0.05	0.61	0.03	0.75	−0.16	0.11
ALP	−0.16	0.10	0.13	0.18	0.04	0.70	0.10	0.33
NLR	−0.08	0.45	0.10	0.28	0.16	0.11	−0.11	0.26
**Post-RT** **Parameters**	**Neurotensin**		**β-Endorphin**		**TRPV1**		**Orexin A**	
	**r**	***p***	**r**	***p***	**r**	***p***	**R**	***p***
NEU	−0.02	0.90	0.14	0.26	−0.05	0.71	0.07	0.57
LEU	0.21	0.09	−0.11	0.41	−0.07	0.57	−0.06	0.65
LDH	0.06	0.62	−0.08	0.51	0.09	0.50	0.04	0.75
ALP	−0.04	0.78	−0.03	0.80	−0.01	0.95	0.12	0.36
PLT	0.00	0.98	0.02	0.86	−0.15	0.23	0.09	0.49
NLR	−0.27	0.03	0.24	0.053	0.01	0.92	0.05	0.67

No significant correlation was found between RT-related parameters and pain-related biomarkers (Table 5).

**Table 5:** Radiotherapy parameters and post-treatment biomarkers.

Radiotherapy Parameters	Post-RT Biomarkers							
	Neurotensin		β-Endorphin		TRPV1		Orexin A	
	r	*p*	r	*p*	r	*p*	r	*p*
Interval days	0.03	0.83	−0.14	0.25	0.14	0.28	0.12	0.34
Daily dose	−0.17	0.18	−0.05	0.67	0.03	0.81	0.04	0.79
Total dose	0.12	0.34	−0.05	0.67	0.10	0.45	0.07	0.58
GTV volume	−0.23	0.06	−0.04	0.75	−0.05	0.68	−0.06	0.61
Min dose	0.21	0.10	0.00	0.99	0.09	0.47	−0.03	0.80
Max dose	−0.07	0.61	−0.03	0.81	−0.04	0.75	0.04	0.75
Mean dose	0.19	0.14	−0.15	0.25	0.08	0.51	−0.14	0.26
Dose rate	−0.22	0.08	−0.14	0.27	−0.10	0.43	0.03	0.84
MU	0.02	0.85	−0.03	0.79	0.02	0.88	−0.03	0.79

The follow-up period of the patients was 5–179 days, 70.2% of the patients had poor response, 63.6% had moderate response, and 80.4% had good response. Patients with a good response had a higher survival rate (Table 1).

## 4. Discussion

There are several basic scales for palliation and monitoring of cancer pain, effective pain management, and improving the patient’s quality of life [16]. Standardized pain scales such as NRS, VAS, and VRS are used to understand the intensity of pain, and pain biomarkers are used to understand the underlying mechanisms of pain. ‘Patient-reported Outcomes’ (PROs), which collect direct feedback from patients about their pain experiences and treatment effects, enable personalization of treatment strategies [17,18]. In addition to standardized scales and patient-derived insights, new scales based on feedback can be developed. In fact, the development of these scales according to the sociocultural characteristics of the patients makes it easier for patients to express pain and pain regression score. We developed a revised pain and response scale that allows patients applying to our clinic to express pain grading and treatment response with an easier and more practical solution (Figure 1). Thanks to the RPS scale, three categories were created at the top of the scale for “pain intensity” before treatment: minimal, moderate, and severe pain. Three categories were also created at the bottom of the scale for “pain response” after treatment: poor, moderate, and good response. This makes it easy to express pain intensity and response using numbers or verbal descriptions. Thus, by integrating subjective pain assessments expressed by patients with objective biochemical assessments, it was thought that cancer pain treatment could be better monitored, interventions could be adjusted, and patient outcomes could be improved. In our study, we found a higher rate of ‘good response’ to RT in patients with breast and prostate cancer. Reducing bone metastasis pain with RT tends to result in higher response rates in patients with breast cancer or prostate cancer compared to patients with lung cancer or other primary cancers [10]. This increased efficacy can be attributed to several factors, including the biological characteristics of tumors, their sensitivity to radiation, and the general health and performance status of patients.

In general, patients with fewer bone metastases and without previous RT benefit more from treatment [19]. The previous RT mentioned is the second serial irradiation applied to the same site. In our study, we did not perform a second series of irradiations; previous RT refers to RT previously applied to the primary cancer site or any other site. As far as we can see, this issue has not been investigated in detail. Marazzi et al. [20] state that if a patient whose primary cancer site has been irradiated is irradiated because of bone metastases, the application of RT may be significantly affected by previous experience. In addition, if bone metastases are located in or near the previously irradiated area, there is a higher risk of developing complications such as radiation-induced fractures, soft tissue damage, or myelopathy. Our study did not find a relationship between previous RT and response score.

The presence of PNI, vertebral, extremity, or mixed localization of metastasis was not found to differ in terms of pain response. When comparing all morphological features of metastasis, including lytic, destructive, soft-tissue, and PET-CT lesions with only PET-CT uptake and no equivalent on conventional imaging, the good response score was higher in sclerotic lesions. Bone metastases may present as osteolytic (osteoclastic), sclerotic (osteoblastic), or mixed lesions, depending on the underlying mechanism of bone involvement. Sclerotic metastases are usually associated with prostate cancer and some types of breast cancer. Sclerotic bone metastases respond better to RT, especially for pain relief and to control local tumor growth, leading to further sclerosis or stabilization of the lesion [21].

In our study, no significant correlation was found between SUVmax values on PET/CT and treatment-related pain response scores. However, SUVmax values of bone metastases in patients with poor pain response were higher than those with good response. Again, the poor pain response score was higher in patients with SUVmax values above 10. In the literature, the correlation between SUVmax and pain intensity is statistically significant, and higher SUVmax values indicate higher pain levels [22,23].

For painful bone metastases, different RT fractionation schemes are used, each designed to provide effective analgesia, taking into account the patient’s overall health status and treatment goals [24]. The decision regarding the fractionation scheme should be based on individual patient-related factors such as the extent of metastasis, general health status, and the presence of concurrent therapies. In our study, the most used fractions were 4×5Gy, 5×5Gy, and 10×3Gy, and there was no relationship between fractions and pain response score. While 4×5Gy and 5×5Gy were used in oligometastases and small RT fields, the 10×3Gy scheme was used in very large, destructive, soft tissue multiple metastases. In this way, effective palliation can be achieved with an appropriate, effective, and intelligent RT scheme.

Biological markers such as TRPV1, bEP, NT, OXA, cytokines, and inflammatory mediators can be measured to provide objective information on pain levels and inflammatory response [25]. These pain markers would be useful in combination with treatment modalities such as radiotherapy, in addition to monitoring pain. In fact, in a biological context, some synthesized complex molecules have shown significant therapeutic effects in vivo in the treatment of tumor-associated osteolytic lesions caused by bone metastases [26].

Activation of TRPV1 leads to the development of metastatic bone pain, release of proinflammatory substances, and sensitization of nociceptive neurons, which may exacerbate bone metastasis-induced pain. The efficacy of RT on bone metastases may influence the overall pain experience in patients due to modulation of TRPV1 expression and activity. Other studies emphasize that TRPV1 antagonists may improve the efficacy of RT by altering pain signaling and immune responses [27]. In our study, we observed that TRPV1 increased significantly in patients with a good response.

Acting as an endogenous analgesic, bEP plays a role in the regulation of pain perception in the body. RT may affect bEP levels by reducing tumor burden, and it is thought that administration of bEP during RT or increasing its levels in the body may help reduce pain perception and potentially complement conventional treatments such as RT [28]. Conversely, there are also studies showing that RT side effects can be improved by utilizing endogenous systems [29]. Using a transgenic mouse model with cancer, as a result of an experimental study investigating how cancer progression and pain perception associated with bone metastases are affected, it has been observed that bEP levels significantly increased after radiation therapy. It has been claimed that bEP may play a role in enhancing the effectiveness of RT [30]. We found that bEP values significantly decreased. The tumor burden decreased with RT, as did pain and bEP concurrently.

It is stated that NT plays a role in the development and progression of bone metastases, which promote tumor growth and angiogenesis, and thus may worsen the overall prognosis of patients with metastatic disease [10]. There is a potential connection between NT levels and RT efficacy in patients with bone metastases. Rising NT may affect RT response associated with increased sensitivity to pain. [31]. In our study, NT decreased after RT in patients with a good response.

OXA, a neuropeptide produced by the hypothalamus, plays an important role in regulating appetite, sleep, and arousal. The relationship between OXA and RT treatment in patients with bone metastases is a newly emerging research area. There is new evidence that OXA can regulate the tumor microenvironment and may potentially enhance the effectiveness of RT [32]. In our study, no significant relationship was found between OXA and pain response score.

There is a significant relationship between NLR and RT efficacy in patients with bone metastasis [33]. A high NLR may reflect a systemic inflammatory response that can affect RT efficacy and side effects [34]. It has been shown that patients with high NLR may have less favorable outcomes from RT [35]. In our study, the pre-RT NLR was higher in patients with a poor response (indicating a higher tumor burden and a more intense immune response), while it was lower in those with a good response. The post-RT NLR was high in patients with a good response but lower than in patients with a poor response: this effect after RT can be attributed to the resulting lymphopenia. Leu values were found to be statistically significantly lower after RT for all groups compared to pre-RT. This was thought to be related to the immune response to RT.

The study’s key limitations include its failure to consider critical biochemical interactions that influence pain transmission and response modulation. Pain perception is complex, involving a blend of physiological processes, sensory input, emotions, and cognitive factors. Biochemical factors, such as neurotransmitters like substance P and neuropeptides like encephalins, play significant roles in pain signaling pathways and overall pain experiences, highlighting a gap in the study’s comprehensive understanding of pain mechanisms. Additionally, the small sample size and lack of a homogeneous patient group raise concerns about the generalization of the findings. Variability among participants regarding genetic backgrounds, comorbidities, and psychological factors can confound results. Future research should focus on integrating biochemical, physiological, and psychosocial perspectives, as a more extensive and diverse cohort could lead to improved insights into chronic pain mechanisms and potentially enhance treatment strategies. Understanding these complex interactions is essential for developing effective approaches to pain management.

## 5. Conclusions

Monitoring, scoring, and quantifying pain are critical for effective pain management, and developing simplified pain rating scales can significantly enhance patient communication. Commonly used multi-step scales enable patients to express their pain levels in a comprehensible manner across all patient groups experiencing pain. However, in our study, our clinical observation was that patients with widespread disease had difficulty selecting from 0- to 10-point scales and were more willing to prefer a simpler, easier-to-understand pain response scale. Thus, the pain response rating scale we developed (poor, moderate, and good) simplified the expression of pain in three steps. The subjective pain scores expressed by patients can be combined with objective biochemical assessments, making it possible to monitor and intervene in cancer pain treatment. In our study, metastases of breast and prostate cancer, sclerotic metastases, oligometastases, and the absence of extra-bone organ metastases increased good response rates.

Initially, a high pain score with high NLR levels was found to have a higher poor response. After treatment, Leu had significantly decreased in all patients regardless of pain response score. In patients with poor response, pre-treatment NT was high, while bED and TRPV1 were low. In patients with a good response, post-treatment NT and bEP decreased, while TRPV1 increased. Our study may contribute to improving treatment outcomes by combining RT with therapies that regulate biomarker activity, especially NT, bED, and TRPV1. Thus, changes in pain management strategies may occur through the use of the body’s own endogenous systems in combating bone metastases and alleviating pain. Nevertheless, we believe that a more comprehensive investigation of tumors, treatment, and biochemical interactions is necessary for the optimization of pain treatment in the future.

## Figures and Tables

**Table 1:** Patient, tumor, and treatment response data.

General Parameters			Pain Response After Radiotherapy			
			Poor Response (*n* = 47)	Moderate Response (*n* = 11)	Good Response (*n* = 47)	*p*
Age	Mean ± SD		63.1 ± 13.1	66.1 ± 14.5	67.7 ± 12.2	0.243
	Median (min–max)		(37–87)	(35–87)	(46–85)	
Gender	Male		30 (63.8)	8 (72.7)	25 (53.2)	0.380
	Female		17 (36.2)	3 (27.3)	22 (46.8)	
Diagnosis	Prostate cancer		8 (17.0)	0 (0.0)	15 (31.9)	0.043
	Breast cancer		12 (25.5)	2 (18.2)	17 (36.2)	
	Lung cancer		12 (25.5)	5 (45.5)	7 (14.9)	
	Other		15 (31.9)	4 (36.4)	8 (17.0)	
Histopathological cancer type	Adenocarcinoma		22 (46.8)	4 (36.4)	21 (44.7)	0.796
	Squamous cell carcinoma		4 (8.5)	1 (9.1)	2 (4.3)	
	Neuroendocrine carcinoma		2 (4.3)	1 (9.1)	2 (4.3)	
	Infiltrative ductal carcinoma		12 (25.5)	2 (18.2)	17 (36.2)	
	Other		7 (14.9)	3 (27.3)	5 (10.6)	
Differentiation	Unknown		24 (51.1)	9 (81.8)	20 (42.6)	0.098
	Good		0 (0.0)	0 (0.0)	2 (4.3)	
	Moderate		6 (12.8)	0 (0.0)	12 (25.5)	
	Poor		17 (36.2)	2 (18.2)	13 (27.7)	
Perineural invasion	Unknown		33 (70.2)	11(100.0)	28 (59.6)	
	No		7 (14.9)	0 (0.0)	7 (14.9)	0.112
	Yes		7 (14.9)	0 (0.0)	12 (25.5)	
Second primary cancer	No		40 (85.1)	10 (90.9)	38 (80.9)	0.680
	Yes		7 (14.9)	1 (9.1)	9 (11.1)	
Pain intensity	Unknown		0 (0.0)	2 (18.2)	4 (8.5)	0.001
	Mild		6 (12.8)	0 (0.0)	9 (19.1)	
	Moderate		16 (34.0)	0 (0.0)	23 (48.9)	
	Severe		25 (53.2)	9 (81.8)	11 (23.4)	
Metastasis location	Appendicular		6 (12.8)	1 (9.1)	11 (23.4)	0.561
	Axial		17 (36.2)	4 (36.4)	12 (25.5)	
	Mixt		24 (51.1)	6 (54.5)	24 (51.1)	
Metastasis type	Increased FDG activity		1 (2.1)	1 (9.1)	9 (19.1)	0.001
	Soft tissue-lytic-destructive-		9 (19.1)	6 (54.6)	4 (8.5)	
	Lytic		12 (25.5)	3 (27.3)	6 (12.8)	
	Mixt		11 (23.0)	1 (9.1)	4 (8.5)	
	Sclerotic		14 (29.8)	0 (0.0)	24 (51.1)	
Number of bone metastases	Multiple		31 (66.0)	8 (72.7)	24 (51.1)	0.223
	Oligo		16 (34.0)	3 (27.3)	23 (48.9)	
Extra-bone metastasis	Unknown		4 (8.5)	0 (0.0)	0 (0.0)	0.089
	Yes		27 (57.4)	6 (54.5)	21 (44.7)	
	No		16 (34.0)	5 (45.5)	26 (55.3)	
Time between diagnosis and month of metastasis	<3		23 (48.9)	9 (81.8)	29 (61.7)	0.670
	3–11		8 (17.0)	0 (0.0)	6 (12.8)	
	12–35		4 (8.5)	1 (9.1)	3 (6.4)	
	36–59		6 (12.8)	0 (0.0)	5 (10.6)	
	≥60		6 (12.8)	1 (9.1)	4 (8.5)	
Parameters related to PET/CT SUVmax						
Primary tumor SUVmax	Median (min–max)		3.9 (0.0–48.4)	6.8 (0.0–19.6)	5.7 (0.0–65.5)	0.500
	<2		17 (36.2)	2 (18.2)	13 (27.7)	0.654
	2–10		19 (40.4)	7 (63.6)	22 (46.8)	
	>10		11 (23.4)	2 (18.2)	12 (25.5)	
Lymph node metastasis SUVmax	Median (min–max)		2.7 (0.0–59.7)	1.9(0.0–10.6)	1.7(0.0–97.3)	0.916
	<2		23 (48.9)	6 (54.5)	24 (51.1)	0.889
	2–10		15 (31.9)	4 (36.4)	17 (36.2)	
	>10		9 (19.1)	1 (9.1)	8 (12.8)	
Bone metastasis SUVmax	Median (min–max)		9.7 (0.0–39.2)	6.3 (3.7–22.5)	9.2(0.0–38.6)	0.646
	<2		2 (4.3)	0 (0.0)	3 (6.4)	0.704
	2–10		22 (46.8)	7 (63.6)	26 (55.3)	
	>10		23 (48.9)	4 (36.4)	18 (38.3)	
Parameters associated with radiotherapy						
Previous RT	Any previous RT	Unknown	2 (4.3)	0 (0.0)	1 (2.1)	0.435
		Yes	18 (38.3)	2 (18.2)	12 (25.5)	
		No	27 (57.4)	9 (81.8)	34 (72.3)	
	Previous primary RT	Unknown	2 (4.3)	0 (0.0)	1 (2.1)	0.179
		Yes	14 (29.8)	1 (9.1)	6 (12.8)	
		No	31 (66.0)	10 (90.9)	40 (85.1)	
	Time between previous RT and current RT (months)	Median (min–max)	29.0 (0–110)	23 (13–33)	45 (0–100)	0.778
RT interval (days)		1–5	22 (46.8)	5 (45.5)	18 (38.3)	0.754
		6–9	12 (25.5)	2 (18.2)	13 (27.7)	
		10–30	13 (27.7)	4 (36.4)	14 (29.8)	
		>30	0 (0.0)	0 (0.0)	2 (4.3)	
RT daily dose (Gy)		2 Gy	0 (0.0)	0 (0.0)	1 (2.1)	0.807
		3 Gy	8 (17.0)	3 (27.3)	11 (23.4)	
		4 Gy	1 (2.1)	0 (0.0)	2 (4.3)	
		≥5 Gy	38 (80.9)	8 (72.7)	33 (70.2)	
Total RT dose (Gy)		20 Gy	6 (12.8)	0 (0.0)	3 (6.4)	0.534
		25Gy	33 (70.2)	8 (72.7)	32 (68.1)	
		≥30	8 (17.0)	3 (27.3)	12 (25.5)	
RT target volume: Gros tumor volume (cm^3^)		<20	5 (10.6)	1 (9.1)	7 (14.9)	0.416
		20–50	9 (19.1)	1 (9.1)	5 (10.6)	
		51–100	5 (10.6)	1 (9.1)	7 (14.9)	
		101–500	16 (34.0)	6 (54.5)	24 (51.1)	
		>500	12 (25.5)	2 (18.2)	4 (8.5)	
Target-covering RT Dose %	Minimum dose	<50	0 (0.0)	1 (9.1)	0 (0.0)	0.131
		50–84	7 (14.9)	1 (9.1)	2 (4.3)	
		85–95	25 (53.2)	5 (45.5)	28 (59.6)	
		95–100	13 (27.7)	4 (36.4)	15 (31.9)	
		>100	2 (4.3)	0 (0.0)	2 (4.3)	
	Maximum dose	100–110	42(89.4)	9 (81.8)	43 (91.5)	0.640
		>110	5 (10.6)	2 (18.2)	4 (8.5)	
	Mean dose	≤100	7 (14.9)	0 (0.0)	9 (19.1)	0.281
		>100	40 (85.1)	11 (100.0)	38 (80.9)	
RT Dose rate		400	10 (25.0)	2 (18.2)	10 (25.6)	0.988
		600	30 (75.0)	9 (81.8)	29 (74.4)	
RT MU		300–600	2 (4.3)	0 (0.0)	5 (10.6)	0.408
		601–1000	11 (23.4)	2 (18.2)	10 (21.3)	
		1001–2000	23 (48.9)	4 (36.4)	17 (36.2)	
		2000–4000	4 (8.5)	3 (27.3)	11 (23.4)	
		>4000	7 (14.9)	2 (18.2)	4 (8.5)	
RT technique		3DCRT	16 (34.0)	4 (36.4)	15 (38.5)	0.912
		IMRT	31 (66.0)	7 (63.6)	29 (61.7)	
Response status in follow-up						
Follow-up period (Month) Mean ± SD			42.1 ± 39.0	16.9 ± 20.4	36.4 ± 39.3	0.080
Median (min-max)			32 (5–130)	9 (5–67)	18 (5–179)	
Survival rate			70.2%	63.6%	80.4%	0.346

RT: Radiotherapy, Gy: Gray, MU: Monitor Unit, IMRT: intensity-modulated radiation therapy, 3DCRT: 3D conformal radiation therapy, FDG: fluorodeoxyglucose.

**Table 2:** Relationship between blood count and biochemical parameters, and treatment response.

Blood Count and Biochemical Parameters		Pain Response After Radiotherapy			
		Poor Response (*n* = 47)	Moderate Response (*n* = 11)	Good Response (*n* = 47)	*p*
Pre-RT Neu	Median	4.6 (1.5–19.1)	5.0 (1.6–10.7)	3.7 (1.4–11.7)	0.116
Post-RT Neu	Median	3.9 (0.8–25.0)	4.7 (1.4–7.8)	3.0 (0.7–8.0)	0.010
*p*		0.006	0.131	<0.001	
Pre-RT Leu	Median	1.2 (0.2–2.7)	1.4 (0.5–4.0)	1.3 (0.3–3.3)	0.439
Post-RT Leu	Median	0.9 (0.2–4.7)	0.9 (0.3–1.4)	0.8 (0.3–3.5)	0.426
*p*		<0.001	0.003	<0.001	
Pre-RT NLR	Median	4.0 (1.3–36.7)	3.3 (0.7–12.1)	2.6 (1.2–11.4)	0.036
Post-RT NLR	Median	3.9 (0.8–37.2)	5.5 (1.5–18.0)	3.1 (1.2–10.8)	0.047
*p*		0.005	0.021	0.002	
Pre-RT PLT	Median	259 (69–528)	374 (190–542)	271 (130–686)	0.105
Post-RT PLT	Median)	204.0 (39–505)	260.0 (72–434)	202.0 (57–554)	0.486
*p*		0.001	0.021	<0.001	
Pre-RT LDH	Median	221.0 (19–1160)	175.0 (113–545)	220.0 (126–642)	0.201
Post-RT LDH	Median	234.0 (99–1064)	188.0 (56–423)	211.0 (117–904)	0.200
*p*		0.202	0.722	0.553	
Pre-RT ALP	Median	98.0 (35–396)	125.0 (81–277)	94.0 (54–1025)	0.374
Post-RT ALP	Median	98.0 (27–528)	121.0 (69–673)	88.0 (26–1002)	0.333
*p*		0.240	0.285	0.065	
Pre-RT tumor markers	Unknown	20 (42.6)	3 (27.3)	8 (17.0)	0.082
	Normal	15 (31.9)	4 (36.4)	17 (36.2)	
	High	12 (25.5)	4 (36.4)	22 (46.8)	

**Table 3:** Relationship between biomarkers and treatment response.

Biochemical Pain Biomarkers		Pain Response After Radiotherapy			
		Poor Response (*n* = 47)	Moderate Response (*n* = 11)	Good Response (*n* = 47)	*p*
Neurotensin	Pre-RT	609.0 (1.0–2452.0)	369.9 (116.6–920.0)	631.4 (39.7–2863.0)	0.057
	Post-RT	480.0 (100.1–2500.0)	671.0 (75.1–1195.0)	400.3 (79.1–1479.0)	0.828
	*p*	0.600	0.176	0.006	
β-Endorphin	Pre-RT	85.5 (32.9–142.8)	71.6 (25.9–115.5)	92.1 (18.7–228.8)	0.514
	Post-RT	36.0 (5.2–200.0)	36.8 (20.2–58.6)	49.1 (13.3–135.6)	**0.031**
	*p*	<0.001	0.018	<0.001	
Transient Receptor Potential Cation Channel Subfamily V-Member 1	Pre-RT	309.6 (25.2–1047.1)	269.1 (78.5–517.5)	321.7 (48.1–1100.7)	0.496
	Post-RT	355.6 (86.4–1473.7)	450.8 (332.6–821.7)	352.8 (119.3–1510.9)	0.238
	*p*	<0.001	0.018	<0.001	
Orexin A	Pre-RT	1389.0 (521.0–3311.0)	1381.0 (602.0–2076.0)	1449.0 (492.0–3311.0)	0.721
	Post-RT	1544.0 (492.0–3311.0)	1675.0 (510.0–2439.0)	1589.5 (694.0–3703.0)	0.947
	*p*	0.202	0.237	0.125	

## Data Availability

The data that support the findings of this study are available by contacting the corresponding authors.
